# A Numerical Investigation of the Thermal Stresses of a Planar Solid Oxide Fuel Cell

**DOI:** 10.3390/ma9100814

**Published:** 2016-09-30

**Authors:** Paulina Pianko-Oprych, Tomasz Zinko, Zdzisław Jaworski

**Affiliations:** Institute of Chemical Engineering and Environmental Protection Processes, Faculty of Chemical Technology and Engineering, West Pomeranian University of Technology, Szczecin 71-065, Poland; tomasz.zinko@zut.edu.pl (T.Z.); zdzislaw.jaworski@zut.edu.pl (Z.J.)

**Keywords:** thermal and residual stresses, planar solid oxide fuel cell (SOFC), computational fluid dynamics (CFD), finite element method (FEM) calculations

## Abstract

A typical operating temperature of a solid oxide fuel cell (SOFC) is quite high above 750 °C and affects the thermomechanical behavior of the cell. Thermal stresses may cause microstructural instability and sub-critical cracking. Therefore, a joint analysis by the computational fluid dynamics (CFD) and computational structural mechanics based on the finite element method (FEM) was carried out to analyze thermal stresses in a planar SOFC and to predict potential failure locations in the cell. A full numerical model was based on the coupling of thermo-fluid model with the thermo-mechanical model. Based on a temperature distribution from the thermo-fluid model, stress distribution including the von Mises stress, shear stress as well as the operating principal stress were derived in the thermo-mechanical model. The FEM calculations were performed under different working conditions of the planar SOFC. The highest total stress was noticed at the lower operating voltage of 0.3 V, while the lowest total stress was determined at the voltage of 0.7 V. The obtained stress distributions allowed a better understanding of details of internal processes occurring within the SOFC and provided helpful guidance in the optimization of a new SOFC design.

## 1. Introduction

Thermal stress analysis is an important method to study the performance of a system operating at high temperatures typical for solid oxide fuel cell (SOFC) working conditions. High temperature may cause damage of a fuel cell due to stresses induced by differences in mechanical and thermal properties of the SOFC materials. A significant effort has been made by many researchers to investigate several materials composing anodes, cathodes, electrolytes, interconnectors as well as sealants in order to evaluate the probability of survival of the cell components. The thermally induced stress–strain behavior of the cell, sealant, and frame components was investigated by Weil et al. [1], Jiang et al. [2], Nakajo et al. [3], Sanjay [4], Fan et al. [5], and Peksen [6]. Weil et al. [1] investigated the magnitudes of thermally induced stress, strain, and part deflection in the cell, seal, and window frame components of a planar SOFC of a bonded compliant seal design with a 50% mismatch in coefficients of thermal expansion (CTE) under uniform heating and cooling conditions. Some bending results during the cooling down, and the stress is transferred to the sealant foil and soft silver braze.

Effects of the cell voltage and temperature non-uniformity on the thermal stress of the SOFC with the bonded compliant seal design were investigated by Jiang et al. [2]. Numerical results showed that an assumed isothermal SOFC configuration leads to an underestimate of the thermal-stress by 28% for the cell and 37% for the metal frame, in comparison to those in practical operating conditions where the temperature was non-uniform. Authors found a large stress region of the cell near the inlet under the practical operating condition. In this region, the thermal stress was underestimated by 46% with the assumption of a uniform temperature. It was concluded that the dominant factor for the thermal stress was location-dependent. In the low temperature inlet region, both the temperature gradient and the difference between the structure temperature and zero stress temperature dominated. However, in the high temperature outlet region, the effect due to the difference between the structure temperature and the zero stress temperature gradient was more important in comparison to the effect of the temperature gradient, which was less significant. In addition, Jiang et al. [2] noticed that, with a lower voltage, the thermal stress of the cell was relatively lower, while the contribution of the temperature gradient to the thermal stress was higher.

That electrochemical degradation and creep of the stacked, single repeating unit components and shrinkage of the anode support affect the mechanical reliability of SOFCs under practical system operating conditions was widely discussed by Nakajo et al. [3]. Based on the Weibull analysis, it was shown that both anodes and cathodes contributed to the probability of cell failure. The temperature dependence of the CTE mismatch between the cathode and anode governed the evolution of the probability of failure of the cathode. It caused a change in stress from tensile to compressive in the cathode depending upon the temperature, with the most critical range being around 973 K and the threshold temperature being around 1073 K. The zones of the highest tensile stress in the anode at room temperature were subjected in operation to compressive stress due to creep deformation.

Sanjay [4] has numerically examined the influence of the air ratio on profiles of temperature and thermal stress in both co-flow and counter flow configurations of the anode-supported SOFC fueled by syngas operating in steady and transient state modes. It was found that the air ratio helps in maintaining uniform temperature distribution within the cell, especially in the counter flow configuration characterized by higher thermal stress, which was cut down by 3.1%–5.8% by increasing the air ratio from 2.0 to 8.5, respectively. SOFCs with co-flow configuration exhibit better performance in terms of thermal stress and carbon deposition, as well as higher efficiency of 22.58% in comparison to the counter flow configuration.

Fan et al. [5] performed numerical investigation to predict thermal stress for both co- and counter-flow configurations of a planar solid oxide fuel cell as functions of the applied materials CTEs, temperature profiles, and the thicknesses of anodes and electrolytes. The anode was subjected to large tensile stresses, while the electrolyte was subjected to large compressive stresses during the first cooling from the sintering temperature. The large tensile stresses in the anode and the large compressive stresses in the electrolyte relaxed partly when the SOFC operated at a high temperature. Some authors [5] predicted that cracks would appear in the anode structure when the positive electrode–electrolyte–negative electrode structure was cooled to room temperature after the sintering. In addition, it was found that the chemical reduction of NiO to Ni in the porous anode leads to a 20% decrease in absolute stress level.

Recently, thermomechanical stress–strain formulations of fuel cell components was highlighted by Peksen [6]. An extended overview of the proposed literature numerical models was given ranging from a single channel or unit layer up to coupled 3D high end system models describing the complex thermomechanical behavior of SOFCs. Thermomechanical modeling issues related to the geometrical idealization, initial and boundary conditions for the highly coupled fluid, and solid mechanics problems were discussed in detail. It was underlined that, due to the fact that SOFCs operate at high temperatures, the employed material properties need to be implemented as temperature-dependent, because they affect the thermomechanical behavior. Special attention was paid to thermal radiation with and without participating gas, which was omitted in most investigations, while it has a significant effect on the thermal behavior. The author [6] thinks that the geometrical simplification should not be limited to the thermo-fluid analysis, as this influences the structural behavior directly. Nevertheless, a final conclusion of the study [6] was the use of numerical modeling aids in understanding the thermomechanical behavior within solid oxide fuel cells and in increasing their thermomechanical reliability.

Therefore, the objective of the present work was to study thermal stresses in the planar SOFC components using the finite element method (FEM) in order to better understand the details of internal processes occurring within the SOFC design proposed by Bossel [7] and for a superior design of a new fuel cell. In a previous work [8], a comprehensive thermodynamic electrochemical modeling using computational fluid dynamics (CFD) was established and the effect of gas flow on temperature as well as on current density had been investigated under a steady-state mode. The novelty of this paper is its focus on the effects of a temperature profile and the coefficients of thermal expansion (CTEs) mismatch between components on the thermal stresses. The obtained results can be applied as the guide for SOFC materials selection and SOFC structure design in the next stage of fuel cell development led by the project partner within the 7th Framework Programme with the acronym SAFARI.

## 2. Model Description

### 2.1. Geometry and Computational Grid of a Planar SOFC

**Figure 1:**
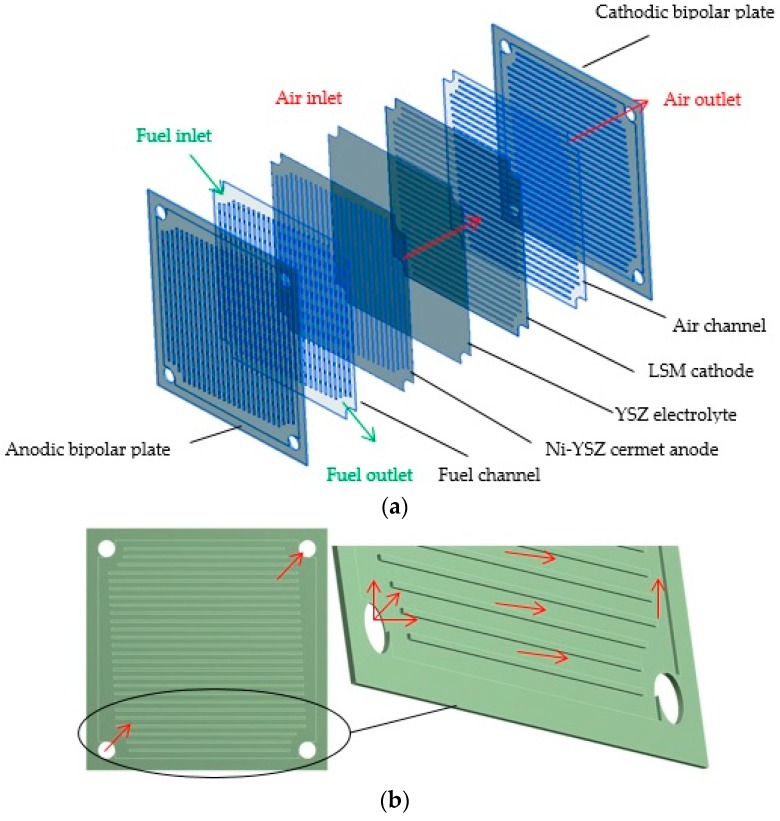
A sketch of a single planar SOFC geometry divided into separate layers with: (**a**) indication of the fluid flow distributions; (**b**) zoom view of the anodic bipolar plate.

The single planar solid oxide fuel cell unit employed in this study is shown in Figure 1, and it consists of (from the left side): a cathodic bipolar plate, an air channel, a LSM (strontium-doped lanthanum manganite) cathode, a YSZ (yttria-stabilized zirconia) electrolyte, a Ni–YSZ cermet anode, a fuel channel, and an anodic bipolar plate. Flow channels were designed by Bossel [7], and the idea was to enable separate flows between two pairs of opposite orifices located at four corners of the bipolar plates. Each of the bipolar ribbed plates had an air channel system on its one side and a fuel channel system on the reverse side. One channel in adjacent plates was used for the air flow along the cathode electrode, while the other one was used for the fuel flow along the anode of each cell. The geometry parameters are presented in Table 1.

**Figure 2:**
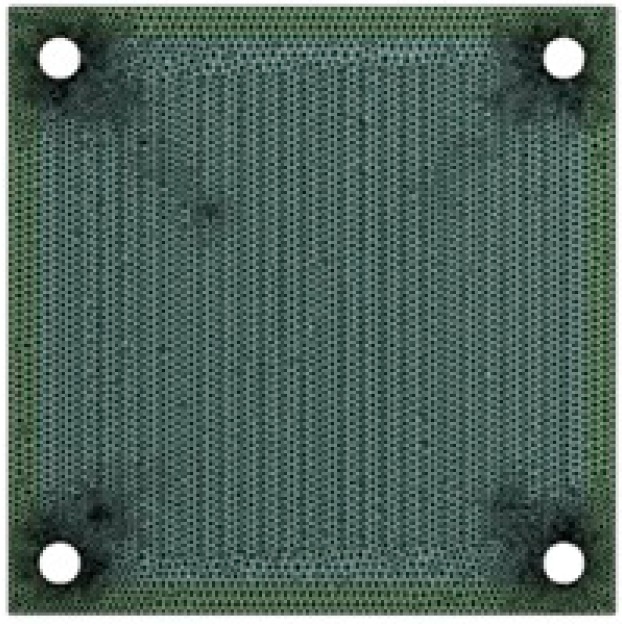
A schematic view of anode and interconnector with a computational grid applied in the finite element method (FEM) calculations.

The numerical grid employed in the CFD simulations consisted of 890 thousand computational cells, while the grid in the FEM simulations had 180 thousand cells. Both types of grids were built in the ANSYS Meshing software. An example of the computational FEM mesh is shown in Figure 2.

### 2.2. Material Properties

Physical properties of the cell components as well as their material properties are presented in Table 2 and Table 3, respectively.

### 2.3. Mathematical Model

The applied thermo-fluid model was explained comprehensively in [8], and the governing equations are summarized in Table 4. The model based on the coupling of balance equations for mass (1), momentum (2), species (3), and energy (4), as well as electronic charge (5) and ionic charge (6), with the electrochemical kinetics of anode and cathode reactions included in the source terms of the governing equations. Thermal energy was transferred by conduction and convection, while the radiative heat transfer was neglected due to its low impact, according to [17].

The mass, species, and momentum conservation equations were solved in the gas channels, and the porous electrodes with the energy equation applied to the entire domain. The ionic charge balance was applied in the anode, electrolyte, and cathode, whereas the electronic charge balance equation was solved in the anode and cathode domains.

The thermo-mechanical model assumed that ceramic cell materials, sealant, and the bipolar plate undergo elastic deformation when subjected to thermal loads. Total strain consisted of elastic and thermal contributions and was defined based on Equation (7):

(7) $$\left\{\varepsilon \right\}=\left\{\varepsilon_{el}\right\}+\left\{\varepsilon_{th}\right\}$$

Thermal strain was calculated from Equation (8):

(8) $$\left\{\varepsilon_{th}\right\}=\left\{\begin{array}{llllll} \alpha & \alpha & \alpha & 0 & 0 & 0 \end{array}\right\}\left(T-T_{ref}\right)$$

where α is the coefficient of thermal expansion (CTE), *T* is the temperature obtained from the thermo-fluid model in the first stage of the CFD simulation, and *T_ref_* is the stress-free temperature.

The stress-strain relationship for an isotropic, linear elastic solid material was computed from Equation (9):

(9) $$\{\begin{array}{c} \sigma_{xx}\\ \sigma_{yy}\\ \sigma_{zz}\\ \sigma_{yz}\\ \sigma_{xz}\\ \sigma_{xy} \end{array}\}=\frac{E}{\left(1+\nu \right)\left(1-2\nu \right)}\left[\begin{array}{cccccc} 1-\nu & \nu & \nu & 0 & 0 & 0\\ \nu & 1-\nu & \nu & 0 & 0 & 0\\ \nu & \nu & 1-\nu & 0 & 0 & 0\\ 0 & 0 & 0 & \frac{\left(1-2\nu \right)}{2} & 0 & 0\\ 0 & 0 & 0 & 0 & \frac{\left(1-2\nu \right)}{2} & 0\\ 0 & 0 & 0 & 0 & 0 & \frac{\left(1-2\nu \right)}{2} \end{array}\right]\{\begin{array}{c} \varepsilon_{xx}\\ \varepsilon_{yy}\\ \varepsilon_{zz}\\ \varepsilon_{yz}\\ \varepsilon_{xz}\\ \varepsilon_{xy} \end{array}\}-\frac{E\cdot \alpha \cdot \Delta T}{1-2\nu }\{\begin{array}{c} 1\\ 1\\ 1\\ 0\\ 0\\ 0 \end{array}\}$$

where *E* is Young’s modulus, and ν is Poisson’s ratio of the modeled material.

The equivalent von Mises stress was described by Equation (10):

(10) $$\sigma_{vM}=\sqrt{\frac{1}{2}\cdot \left[{\left(\sigma_{xx}-\sigma_{yy}\right)}^{2}+{\left(\sigma_{yy}-\sigma_{zz}\right)}^{2}+{\left(\sigma_{zz}-\sigma_{xx}\right)}^{2}\right]+3\cdot \left(\sigma_{xy}^{2}+\sigma_{yz}^{2}+\sigma_{zx}^{2}\right)}$$

Numerical thermo-fluid modeling in the planar SOFC was carried out in the flow solver ANSYS Fluent supported by the Fuel Cell Module. From this first stage of the CFD calculations, local values of the velocity, pressure, temperature, species concentrations, and current density were delivered. The second stage of the modeling was based on temperature distributions imported from the thermo-fluid model into the thermomechanical solver, where computational structural mechanics analysis was performed using the commercial software ANSYS Mechanical module Static Structural. In this stage of the FEM calculations, the stress solver delivered stress distribution including the von Mises stress in ceramic materials.

### 2.4. Boundary Conditions

In order to complete the FEM model formulation, boundary conditions of the single planar SOFC were required. First of all, steady-state processes were considered. In addition, structural constraints with one degree of freedom in the cell axial direction at the outer surface of the cathode bipolar plate was assumed. The reference temperature was equal to 700 °C. The only load in the thermomechanical model stemmed from the working temperature of the fuel cell estimated in the CFD modeling. The impact of gravity was neglected.

In the CFD model, the SOFC was supplied with a mixture of 95 wt % hydrogen and 5 wt % water as well as 23 wt % oxygen and 77 wt % nitrogen at a flow rate of fuel at 4.9 × 10^−8^ (kg/s) and of air at 1.7 × 10^−6^ (kg/s). The anode exchange current density was equal to 7460 (A·m^−2^), whereas that for the cathode was 10,090 (A·m^−2^). The outer current collector surface (anode side) was defined as the voltage tap surface, equal to 0 V, while the current tap surface was defined as the outer current collector surface (cathode side), equal to 0.3, 0.7, or 1.1 V.

To initialize the numerical FEM solutions, the predicted temperature distributions for the anode, electrolyte, cathode, and current collectors were implemented into the thermo-mechanical model to estimate stress distributions in the planar SOFC and to assess the effects of the operating temperature on the thermal elongations. The residual stresses were assumed to be developed during the manufacturing process of the fuel cell. However, instead of assuming two-stage cooling process of the sintered fuel cell layers (anode with electrolyte and then thermal treatment of the anode-electrolyte layers with the cathode), only a one-stage thermal treatment process was considered. Sintering of the fuel cell at 1350 °C and cooling down to room temperature of 25 °C was assumed. The model presented in this paper in the first step included an analysis of residual stresses induced in the anode-electrolyte layers due to the CTE differences. In the calculation of the residual stress, a free stress temperature was set as that of the sintering temperature of the fuel cell layers. The total stresses, including the residual stresses as well as those resulting from temperature distributions, were applied to the FEM model from the CFD in the second step of numerical analysis.

## 3. Simulations Results and Discussion

**Figure 3:**
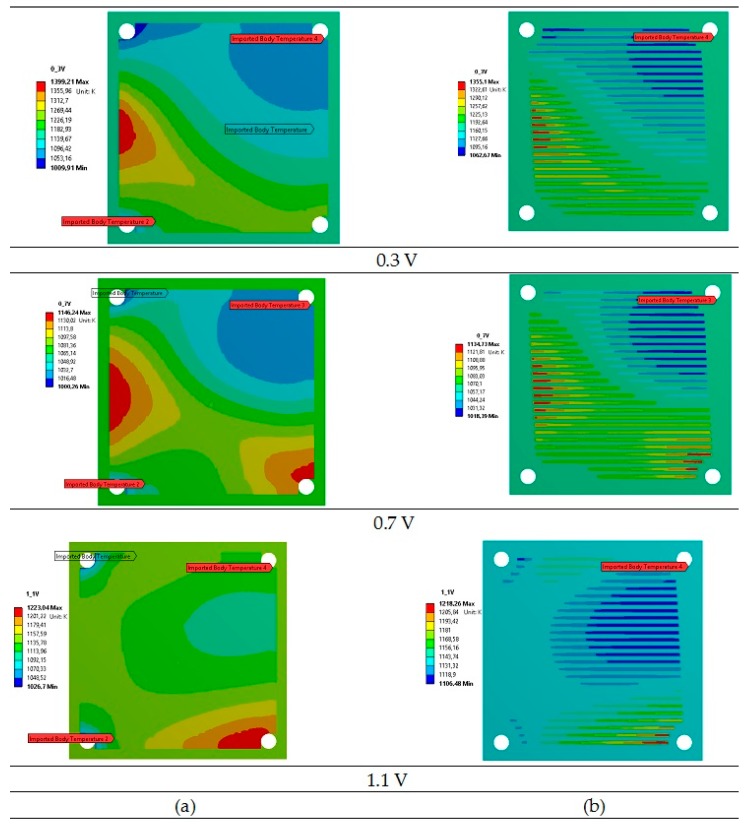
The calculated profiles of temperature imported from ANSYS Fluent into ANSYS Mechanical at chosen voltages of 0.3, 0.7, and 1.1 V at the surfaces of the (**a**) cathode and (**b**) the cathode bipolar plate.

The profiles of temperature in the planar SOFC calculated in the thermo-fluid model and imported into the thermo-mechanical model are shown in Figure 3. It can be seen that temperature increases rapidly between the air inlet located at the upper left corner and the air outlet located at the lower right corner due to the exothermic electrochemical reactions. The average temperature differences were equal to 389 °C, 146 °C, and 196 °C for the current tap voltages of 0.3, 0.7, and 1.1 V, respectively. The highest difference was noticed for the lowest operating voltage of 0.3 V. In addition, the highest temperature of the air was in the region between the air inlet and fuel inlet for the voltages of 0.3 V and 0.7 V, while, for the voltage of 1.1 V, it was in the region close to the air outlet. The temperature distributions were characterized by high non-uniformity, which may cause local thermal stresses and may lead to fuel cell damage. Thus, the next step was to analyze the planar SOFC behavior in terms of stress.

**Figure 4:**
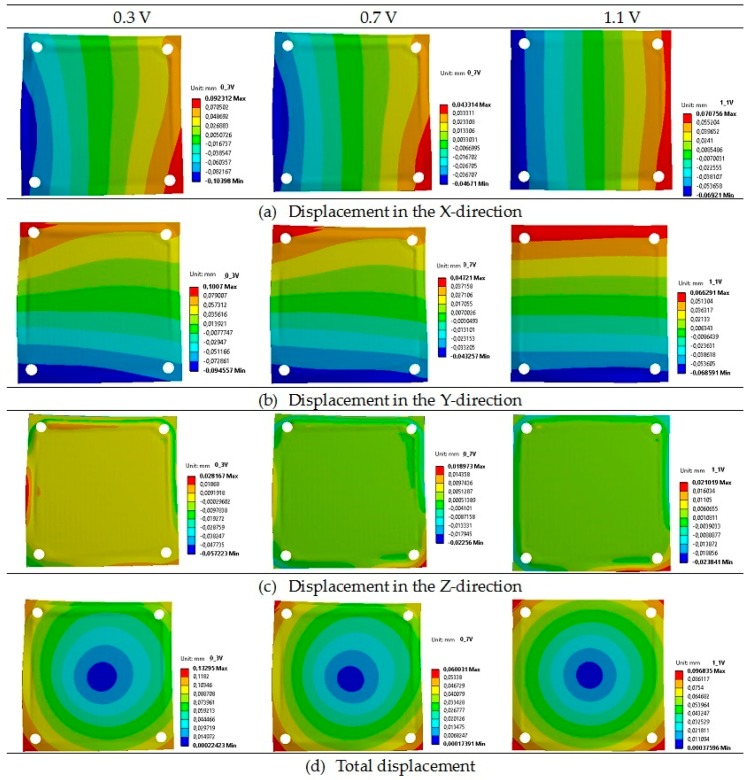
Displacements [mm] of the planar SOFC under thermal stresses (**a**) in the X direction (horizontal); (**b**) in the Y direction (vertical); and (**c**) in the Z direction (perpendicular); (**d**) total displacement at the voltages of 0.3, 0.7, and 1.1 V.

In order to justify the impact of the operational temperature during thermal stresses generation, the simulated deformation distributions in the fuel cell were plotted and are shown in Figure 4.

The lowest total displacement was equal to 0.06 mm, and it was for the voltage of 0.7 V. For the voltage of 1.1 V, it was equal to 0.09 mm. The computed highest total displacement was equal to 0.13 mm for the voltage of 0.3 V.

**Figure 5:**
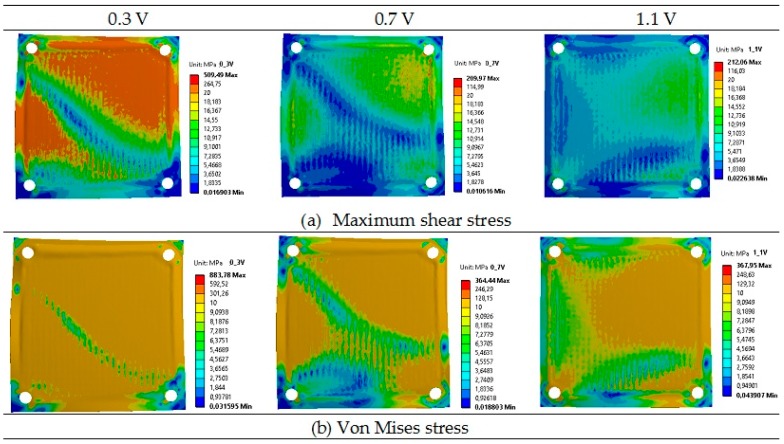
Distributions of the operating maximum shear stress (**a**) and von Mises stress (**b**) of the single planar SOFC MPa.

Figure 5 presents obtained contours of the maximum shear stress and von Mises stress in an operating planar SOFC across the plates. The results reveal that the lowest von Mises stress was equal to 364 MPa for the voltage of 0.7 V, while the highest was obtained for the voltage of 0.3 V and was equal to 884 MPa. For the operational voltage of 1.1 V, the von Mises stress was only slightly higher than the one for the voltage of 0.7 V. Distributions of von Mises stress show the regions with high values that need attention due to the risk of fuel cell damage.

Table 5 presents a comparison of the maximum and minimum principal stresses for anode, electrolyte, and cathode layers in the planar SOFC. Maximum and minimum values are components of the principal stresses. At the operating temperature, the principal stresses were the highest for the electrolyte layers with a tensile value of 509 MPa and a compressive value of −810 MPa at the voltage of 0.3 V, whereas the stress was 219 MPa and −287 MPa for the voltage of 0.7 V, and 277 MPa and −327 MPa for the voltage of 1.1 V.

Lower principal stresses were noticed for the cathode, where the highest stresses were 409 MPa (tensile) and −639 MPa (compressive) for the operational voltage of 0.3 V. Slightly lower principal stresses were found for the anode, where again the highest values were obtained for the voltage of 0.3 V. The calculated values of principal stresses for the anode at 0.7 V and 1.1 V were quite similar. It should be underlined that the lowest principal stresses can be observed in the bipolar plate: the compressive stress of −192 MPa and the tensile stress of 400 MPa, both for the voltage of 0.3 V, are the worst case. The maximum and minimum values of the principal stress are assembled in Table 5. Table 5 shows that the greatest principal stresses were obtained for the voltage of 0.3 V, while the lowest were obtained for the voltage of 0.7 V. In addition, it should be underlined that both the total maximum and minimum principal stresses for all considered layers were tensile at the operational temperature.

**Figure 6:**
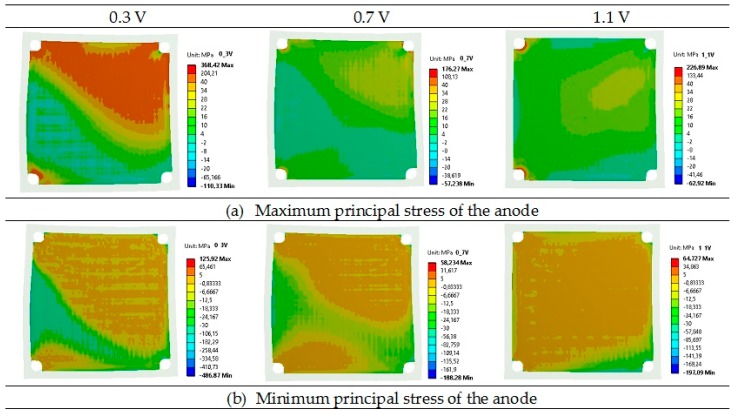
Distributions of the operating maximum (**a**) and minimum (**b**) principal stress of the anode in the single planar SOFC (MPa) for the operational voltages of 0.3, 0.7, and 1.1 V.

**Figure 7:**
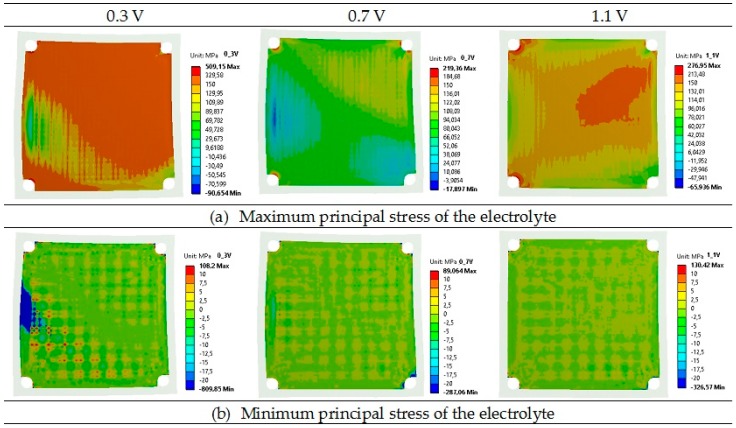
Distributions of the operating maximum (**a**) and minimum (**b**) principal stress of the electrolyte in the single planar SOFC (MPa) for the operational voltages of 0.3, 0.7, and 1.1 V.

**Figure 8:**
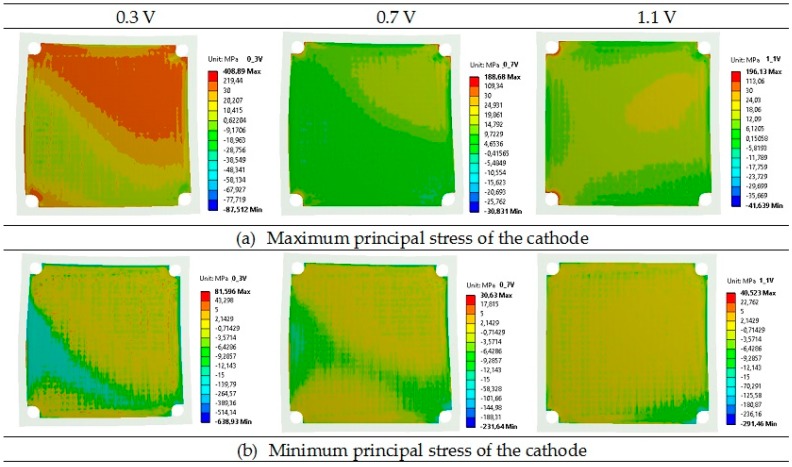
Distributions of the operating maximum (**a**) and minimum (**b**) principal stress of the cathode in the single planar SOFC (MPa) for the operational voltages of 0.3, 0.7, and 1.1 V.

**Figure 9:**
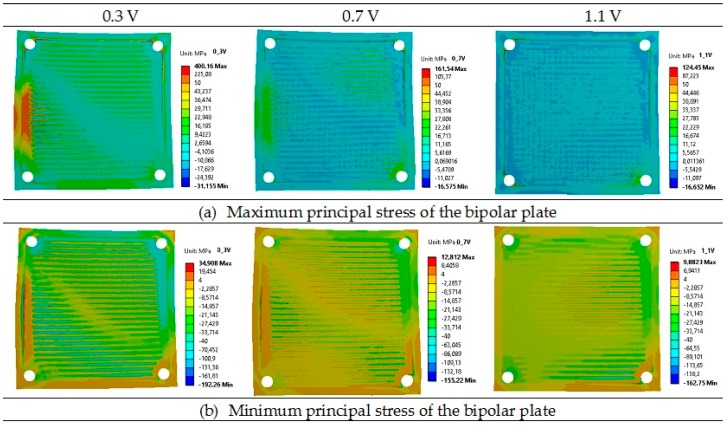
Distributions of the operating maximum (**a**) and minimum (**b**) principal stress of the bipolar plates in the single planar SOFC (MPa) for the operational voltages of 0.31 V, 0.71 V and 1.1 V.

Contour maps of the operating maximum and minimum principal stresses of the anode, electrolyte, cathode, and bipolar plates are presented in Figure 6, Figure 7, Figure 8 and Figure 9.

As shown in Figure 6, Figure 7, Figure 8 and Figure 9, a noticeable increase in the stress values can be observed for all MEA (membrane-electrode assembly) layers and bipolar plate across the fuel cell from its center towards the corners of the cell in both directions.

**Figure 10:**
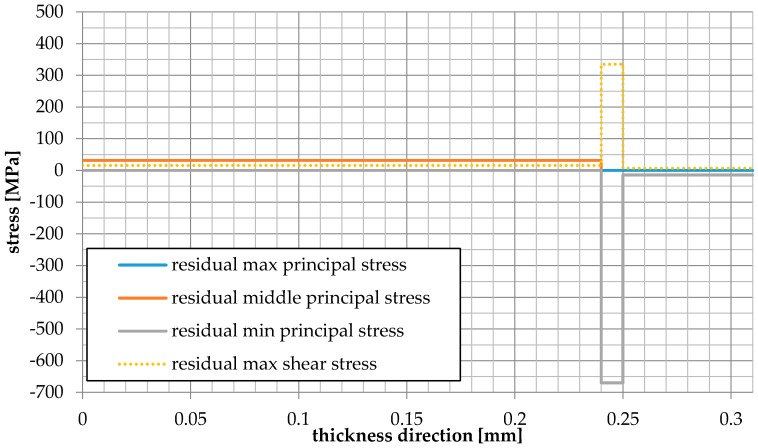
Residual principal stresses and residual shear stress distributions along the thickness of the MEA along the horizontal centerline.

The stress distribution was analyzed by taking into account the residual stresses resulting from manufacturing processes, as shown graphically in Figure 10. It was possible to gain knowledge related to the operating conditions during manufacturing processes and to assess the probability of fuel cell failure. It was found that the residual stresses were significantly higher than the stresses generated during the operational temperature only. The tensile residual stresses occurred in the anode, while the compressive residual stresses were for the cathode and electrolyte. The total stresses were tensile for the anode and compressive for the remaining two layers: cathode and electrolyte.

**Figure 11:**
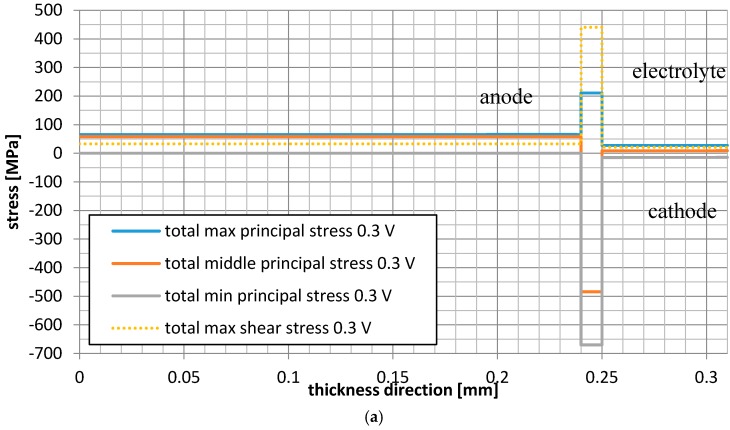

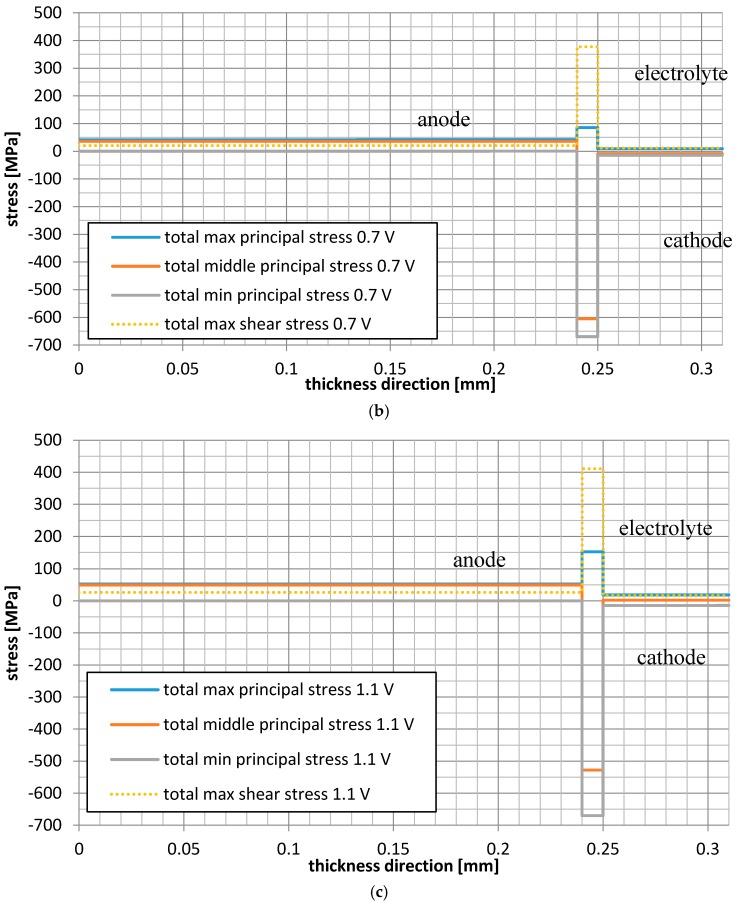
Distributions of the total stresses and total shear stress for the operational temperature and manufacturing processes in the fuel cell along the thickness of the MEA along horizontal centerline for the voltage of : (**a**) 0.3 V; (**b**) 0.7 V; (**c**) 1.1 V.

Moreover, the total principal stresses and the total shear stresses resulted from the operating temperature and manufacturing processes are shown in Figure 11, separately for the operational voltages of 0.3 V, 0.7 V and 1.1 V. The highest compressive total principal stress for the horizontal centerline was found for the electrolyte and it was equal to −670 MPa for all considered voltage values. The total principal stress for the cathode was in the range of −14.5 (compressive) to 15 MPa (tensile), while the highest tensile total principle stress was obtained for the anode and it was equal to 65.5 MPa, 52.0 MPa, and 41.5 MPa for the voltage values of 0.31 V, 0.71 V, and 1.1 V, respectively.

It should be underlined that, for all considered operational voltage values, the minimum total principal stresses were the same (roughly equal to −670 MPa) and depended only on the values of the residual stresses.

**Figure 12:**
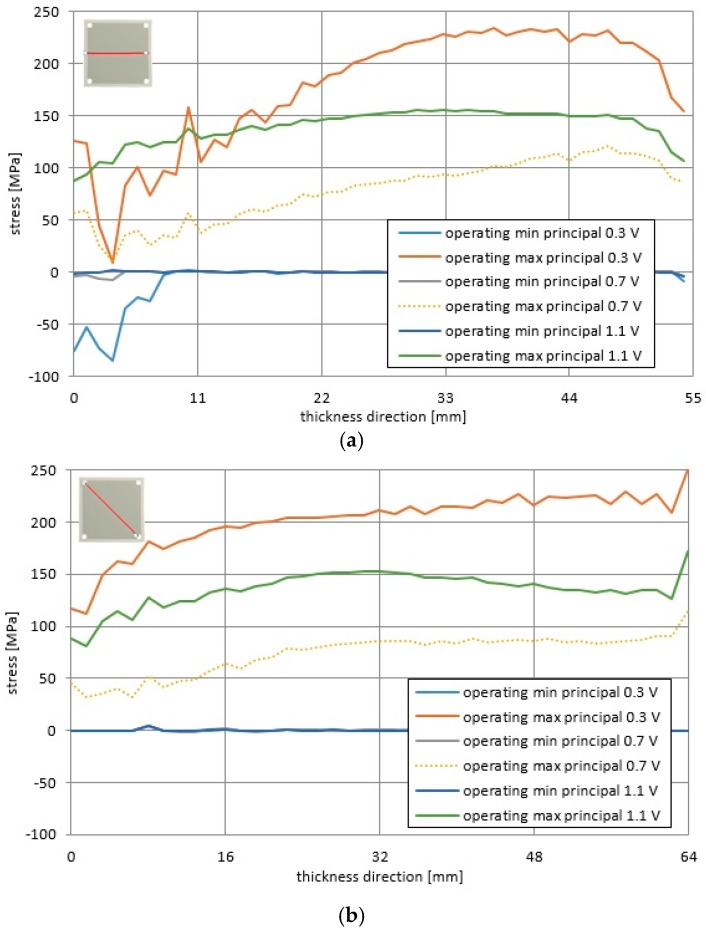
Distributions of the operating principal stresses for the operational temperature in the fuel cell for the voltages of 0.3 V, 0.7 V and 1.1 V along (**a**) the horizontal direction; (**b**) diagonal direction.

**Figure 13:**
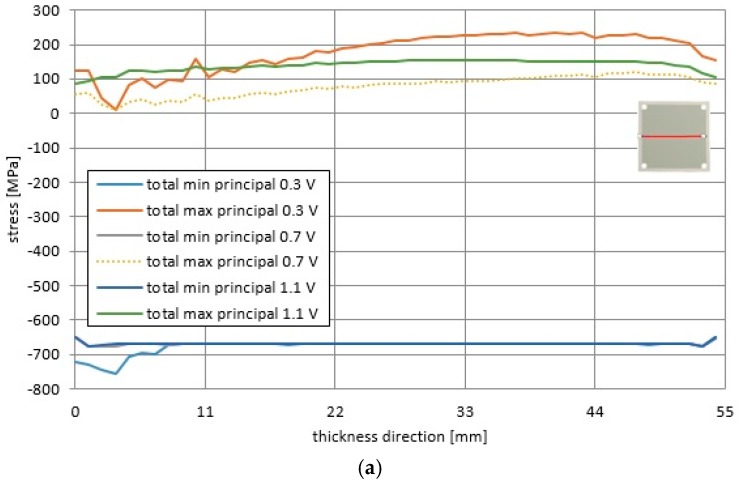

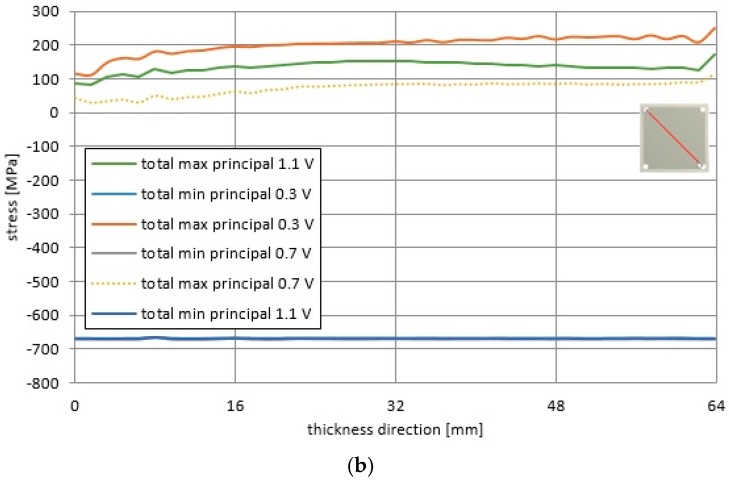
Distributions of the total principal stresses for the operational temperature and accounting for manufacturing processes in the fuel cell for the voltages of 0.3 V, 0.7 V and 1.1 V along (**a**) the horizontal direction; (**b**) the diagonal direction.

In addition, the longitudinal profiles of the total principal stresses and the operational principal stresses presented in Figure 12 and Figure 13 are significantly non-uniform along the horizontal and diagonal directions. Less uniform distributions were obtained for the cell voltage of 0.3 V, but distributions were more uniform for the voltage of 1.1 V. It seems that the stress distributions were more uniform in cases where the cooling air flow was stronger and the probability of fuel cell failure was lower.

In Figure 13, it can be noticed that the total minimum principal stresses for the considered voltage values was roughly equal to −670 MPa, while the total maximum principal stress was equal respectively to 240 MPa, 110 MPa, and 150 MPa for 0.3 V, 0.7 V, and 1.1 V.

## 4. Conclusions

The coupled computational fluid dynamics and computational structural mechanics analysis was performed to investigate thermal stresses in the planar solid oxide fuel cell. Manufacturing processes were modeled to calculate the residual stresses. The residual stresses were treated as initial stresses, and the thermal stresses at the operational temperature were estimated using the temperature distributions imported from the thermo-fluid model.

The impact of different parameters determining the stresses distributions in the SOFC components such as residual stresses, temperature gradients across the fuel cell, and three levels of operational voltage were considered. It was found that both the compressive and tensile stresses can exist in the fuel cell simultaneously. In addition, the simulation results indicate that the highest value of stresses were noticed for the lowest operational voltage value of 0.3 V. Running simulations for three operating points selected in this study (0.3 V, 0.7 V, and 1.1 V) have shown that the terminal voltage of 0.3 V represented significantly abnormal conditions, and such conditions should be avoided due to the highest stresses and a high risk of fuel cell damage.

A critical tensile stress value was recognized for the electrolyte layer at 0.3 V, and it exceeds the tensile yield strength value. Thus, the high risk of failure could probably appear in the electrolyte. Therefore, the electrolyte with a high value for the maximum principal stress needs special attention.

Based on the FEM results obtained in this study, it can be concluded that the operational temperature as well as temperature gradients across the planar solid oxide fuel cell are the main factors that cause the tensile stress in the electrolyte. It is recommended that the electrolyte CTE parameter is better matched with the coefficients of thermal expansion for the anode and the cathode to avoid stresses due to temperature difference, in order to ensure better reliability for the given SOFC design.

## Figures and Tables

**Table 1:** SOFC unit geometry.

Name	Dimension	Unit
Dimensions of fuel cell, bipolar plates, and fuel/air channels	60 × 60	mm^2^
Anode thickness	0.25	mm
Electrolyte thickness	0.01	mm
Cathode thickness	0.06	mm
Thickness of the bipolar plate with gas channel	1	mm
Depth of the flow channels	0.35	mm
Diameter of the fuel and air flow holes	4.2	mm
Active area of the fuel cell	27	cm^2^
Number of channel ribs with a different length	26	-

**Table 2:** Physical properties of cell components.

Name	Dimension	Unit
Exchange current density of anode	7460	A/m^2^
Exchange current density of cathode	10,090	A/m^2^
Anode/cathode porosity	0.3	-
Anode/cathode tortuosity	6	-

**Table 3:** Material properties of cell components [9,10,11,12,13,14,15,16,17].

Name	Anode	Electrolyte	Cathode	Current Collectors
Material	Ni–YSZ	YSZ	LSM	-
Density, kg/m^3^	7740	6000	5300	7450/7700
Specific heat capacity, J/kgK	595	400	607	600
Thermal conductivity, W/mK	6.23	2.7	10	27
Resistivity, Ω·m	-	0.1	-	-
Electronic conductivity, 1/Ω·m	30,300	-	12,800	769,000
Anode-current collectors contact resistance, Ω·m^2^	1 × 10^−7^	-	1 × 10^−8^	-
Coefficient of thermal expansion, 1/K	12.2	10.3	11.7	10.3–12.7
Young’s modulus, GPa	57	215/185	35	214–244
Poison’s coefficient, -	0.28	0.32/0.313	0.36	0.29
Tensile yield strength, MPa	115	332/256	155	291
Compressive strength, MPa	100	1000	100	345
Stress free temperature, K	1623	1623	1473	-

**Table 4:** Governing equations of the thermo-fluid model.

Name	Equation	No
Mass	$\nabla \cdot \left(\rho \bar{v}\right)=R_{j}$	(1)
Momentum	∇⋅(ρv¯v¯)=−∇p+∇⋅[μ(∇v¯+(∇v¯))T]−23∇⋅v¯I+ρg¯	(2)
Species	$\nabla \cdot \left(\rho \bar{v}-\rho x_{i}\sum_{k=1}^{N}D_{i,k}^{eff}\nabla x_{i}\right)=R_{j}$	(3)
**Energy**		
anode	$\begin{array}{l} \nabla \cdot \left(-k\nabla T\right)=\frac{R_{H_{2},anode}T\left(S_{H_{2}O}-0.5S_{O_{2}}-S_{H_{2}}\right)}{M_{H_{2}}}+n_{H_{2},anode}\left|Q_{H_{2},anode}\right|+\\ +\frac{i_{ion}^{2}}{\sigma_{anode,ion}}+\frac{i_{elec}^{2}}{\sigma_{anode,elec}} \end{array}$	(4a)
electrolyte	$\nabla \cdot \left(-k\nabla T\right)=\frac{i_{ion}^{2}}{\sigma_{elec,ion}}$	(4b)
cathode	$\nabla \cdot \left(-k\nabla T\right)=n_{O_{2},cathode}\left|Q_{O_{2},cathode}\right|+\frac{i_{ion}^{2}}{\sigma_{cathode,ion}}+\frac{i_{elec}^{2}}{\sigma_{cathode,elec}}$	(4c)
Inter-connectors	$\nabla \cdot \left(-k\nabla T\right)=\frac{i_{elec}^{2}}{\sigma_{elec-\mathrm{int}ercon}}$	(4d)
Air/fuel channel	$\nabla \cdot \left(-k\nabla T+\rho C_{p}T\cdot \bar{v}\right)=0$	(4e)
**Electronic Charge**		
anode	$-\nabla \cdot \left(\sigma_{elec}^{eff}\nabla \varphi_{elec}\right)=S_{elec,anode}$	(5a)
electrolyte	$-\nabla \cdot \left(\sigma_{elec}^{eff}\nabla \varphi_{elec}\right)=S_{elec,\mathrm{int}er}=0$	(5b)
cathode	$-\nabla \cdot \left(\sigma_{elec}^{eff}\nabla \varphi_{elec}\right)=S_{elec,cathode}$	(5c)
**Ionic Charge**		
anode	$-\nabla \cdot \left(\sigma_{ion}^{eff}\nabla \varphi_{ion}\right)=S_{ion,anode}$	(6a)
electrolyte	$-\nabla \cdot \left(\sigma_{ion}^{eff}\nabla \varphi_{ion}\right)=S_{ion,elec}=0$	(6b)
cathode	$-\nabla \cdot \left(\sigma_{ion}^{eff}\nabla \varphi_{ion}\right)=S_{ion,cathode}$	(6c)

*C_p_*: specific heat; $D_{i,k}^{eff}$: effectiven binary diffusivities; *E_max_*: maximum potential; *g*: gravity; *i*: transfer current density; *I*: identify matrix; *k*: thermal conductivity; *p*: pressure; *R_j_*: volumetric consumption of the j-th species; *S*: source term of the governing equations; *T*: temperature; $\bar{v}$: velocity vector; *V_cell_*: voltage; *x_i_*: species mole fraction; μ: dynamic viscosity; ρ: density; σ: electron/ion conductivity; φ: potential.

**Table 5:** Selected stress results from the thermo-mechanical model.

Stress (MPa)		Anode Ni–YSZ		Electrolyte YSZ		Cathode LSM		Bipolar Plate/ Current Colector Crofer 22 APU	
**Voltage**	Global values in whole part	min	max	min	max	min	max	min	max
**0.3 V**	Maximum principal stress	−110	368	−91	509	−88	409	−31	400
	Minimum principal stress	−487	126	−810	109	−639	82	−192	35
**0.7 V**	Maximum principal stress	−57	176	−18	219	−31	189	−17	162
	Minimum principal stress	−188	58	−287	89	−232	31	−155	13
**1.1 V**	Maximum principal stress	−63	227	−66	277	−42	196	−17	124
	Minimum principal stress	−197	65	−327	130	−291	41	−163	10
